# Developmental and Injury-induced Changes in DNA Methylation in Regenerative versus Non-regenerative Regions of the Vertebrate Central Nervous System

**DOI:** 10.1186/s12864-021-08247-0

**Published:** 2022-01-04

**Authors:** Sergei Reverdatto, Aparna Prasad, Jamie L. Belrose, Xiang Zhang, Morgan A. Sammons, Kurt M. Gibbs, Ben G. Szaro

**Affiliations:** 1 Department of Biological Sciences, University at Albany, State University of New York, Albany, NY 12222 USA; 2 Center for Neuroscience Research, University at Albany, State University of New York, Albany, NY 12222 USA; 3 RNA Institute, University at Albany, State University of New York, Albany, NY 12222 USA; 4 Department of Environmental and Public Health Sciences, University of Cincinnati College of Medicine, Cincinnati, OH 45267 USA; 5 Department of Biology & Chemistry, Morehead State University, Morehead, KY 40351 USA

**Keywords:** *Xenopus laevis*, Spinal cord injury, Optic nerve injury, Axon regeneration, Central nervous system, DNA methylation

## Abstract

**Background:**

Because some of its CNS neurons (*e.g.*, retinal ganglion cells after optic nerve crush (ONC)) regenerate axons throughout life, whereas others (*e.g.*, hindbrain neurons after spinal cord injury (SCI)) lose this capacity as tadpoles metamorphose into frogs, the South African claw-toed frog, *Xenopus laevis*, offers unique opportunities for exploring differences between regenerative and non-regenerative responses to CNS injury within the same organism. An earlier, three-way RNA-seq study (frog ONC eye, tadpole SCI hindbrain, frog SCI hindbrain) identified genes that regulate chromatin accessibility among those that were differentially expressed in regenerative vs non-regenerative CNS [11]. The current study used whole genome bisulfite sequencing (WGBS) of DNA collected from these same animals at the peak period of axon regeneration to study the extent to which DNA methylation could potentially underlie differences in chromatin accessibility between regenerative and non-regenerative CNS.

**Results:**

Consistent with the hypothesis that DNA of regenerative CNS is more accessible than that of non-regenerative CNS, DNA from both the regenerative tadpole hindbrain and frog eye was less methylated than that of the non-regenerative frog hindbrain. Also, consistent with observations of CNS injury in mammals, DNA methylation in non-regenerative frog hindbrain decreased after SCI. However, contrary to expectations that the level of DNA methylation would decrease even further with axotomy in regenerative CNS, DNA methylation in these regions instead increased with injury. Injury-induced differences in CpG methylation in regenerative CNS became especially enriched in gene promoter regions, whereas non-CpG methylation differences were more evenly distributed across promoter regions, intergenic, and intragenic regions. In non-regenerative CNS, tissue-related (*i.e.*, regenerative vs. non-regenerative CNS) and injury-induced decreases in promoter region CpG methylation were significantly correlated with increased RNA expression, but the injury-induced, increased CpG methylation seen in regenerative CNS across promoter regions was not, suggesting it was associated with increased rather than decreased chromatin accessibility. This hypothesis received support from observations that in regenerative CNS, many genes exhibiting increased, injury-induced, promoter-associated CpG-methylation also exhibited increased RNA expression and association with histone markers for active promoters and enhancers. DNA immunoprecipitation for 5hmC in optic nerve regeneration found that the promoter-associated increases seen in CpG methylation were distinct from those exhibiting changes in 5hmC.

**Conclusions:**

Although seemingly paradoxical, the increased injury-associated DNA methylation seen in regenerative CNS has many parallels in stem cells and cancer. Thus, these axotomy-induced changes in DNA methylation in regenerative CNS provide evidence for a novel epigenetic state favoring successful over unsuccessful CNS axon regeneration. The datasets described in this study should help lay the foundations for future studies of the molecular and cellular mechanisms involved. The insights gained should, in turn, help point the way to novel therapeutic approaches for treating CNS injury in mammals.

**Supplementary Information:**

The online version contains supplementary material available at 10.1186/s12864-021-08247-0.

## Background

For over a century, the inability to recover from paralyzing, traumatic injuries to the central nervous system (CNS) has been understood to result from the failure of the damaged axons to regenerate sufficiently to re-establish functional connections [93]. Despite years of intense investigation, the reasons for this incapacity are still only partly understood. The remarkable ability that anamniotes possess to functionally recover from CNS injuries raises the prospect that understanding how these animals do it naturally will provide clues to what needs to happen for mammals to recover. Anuran amphibians (*i.e.*, frogs) occupy a transition point in the phylogenetic, progressive loss of CNS regenerative capacity from anamniote to amniote. Like other anamniotes, frogs regenerate optic axons to restore vision throughout life [10, 101, 109], but like the amniotes, they lose the ability to regenerate spinal cord axons developmentally, during metamorphosis [9, 31, 37]. In both frog and mammal, the loss of CNS regenerative capacity in late development is directly caused by increased exposure to thyroid hormone, which initiates anuran metamorphosis and the final stages of mammalian fetal development [4, 9, 36]. This phylogenetically conserved connection between thyroid hormone production and the hormonally driven, developmental loss of CNS axonal regenerative capacity suggests that in both frog and mammal, the loss involves widespread genetic reprogramming. Indeed, genome-wide expression studies in the hindbrain and spinal cord of *Xenopus laevis* have demonstrated that the response to spinal cord injury (SCI) differs markedly between regenerative and non-regenerative states [11, 36, 64].

In both amniotes and anamniotes, the accessibility of regeneration-associated genes for transcription remains high and even increases with injury in species that can regenerate; in contrast, it becomes developmentally restricted in species that cannot [117, 119, 120]. One mechanism implicated in regulating this accessibility is epigenetic changes in DNA methylation. For example, the methylation state of CpG islands, which are clusters of dinucleotides concentrated within 5mC-depleted domains surrounding transcriptional start sites (TSS), has been linked to both gene activation and repression. Generally, decreases favor higher levels of gene expression and vice versa [reviewed, for example, in [24, 66, 74]. Changes in DNA methylation were first discovered to underlie pervasive changes in gene expression accompanying hormonally driven life-stage transitions in honey bees [43]. Since then, alterations in DNA methylation have been found at life-stage transitions in other animals, including frogs [14]. For example, in *Xenopus*, the activity of the enzyme responsible for *de novo* methylation of cytosines, DNMT3a, increases in response to thyroid hormone, and changes in DNA methylation both accompany and are required for normal metamorphosis [91]. The magnitude of these changes varies across regions of the CNS [58], raising the possibility that such variations may underlie the regional differences in regenerative capacity that arise during metamorphosis in *Xenopus.* In mammals, evidence linking DNA methylation state with axonal regenerative capacity has associated both DNA methylation (5mC) and hydroxymethylation (5hmC) with regenerative success [13, 46, 75, 79, 87, 120, 125, 126, 126]. However, difficulties encountered parsing the relative contributions of DNA methylation states to regenerative success in mammals has left our understanding somewhat ambiguous [13]. Comparing DNA methylation states between regenerative and non-regenerative regions of the CNS in an animal like *Xenopus* should help clarify our understanding.

Our earlier RNA-seq study comparing a region of the CNS before and after the developmental transition from regenerative to non-regenerative stages (tadpole vs. frog hindbrain in spinal cord injury (SCI)) and a region that maintains its regenerative capacity after metamorphosis (frog eye after optic nerve crush (ONC)) has provided indirect evidence implicating DNA methylation states in successful CNS axon regeneration [11]. Of 324 genes that were differentially expressed in successful but not unsuccessful axon regeneration (DESR genes), nine have roles in regulating DNA methylation and hydroxymethylaton, thereby implicating these epigenetic changes in successful axon regeneration. To assess the methylation state of DNA directly, we have now performed comprehensive, Whole Genome Bisulfite Sequencing (WGBS; 15X genome coverage; three biological replicates per condition [133]) on DNA collected from the very same animals used in the earlier RNA-seq study, at the timepoint when differential expression of these genes was greatest. Consistent with expectations that increased DNA methylation should correlate with reduced regenerative potential, DNA methylation levels of non-regenerative, post-metamorphic hindbrain were greater than those of the two regenerative CNS regions (tadpole hindbrain and post-metamorphic eye), and similar to reports in mammalian studies [13, 46], CNS injury decreased overall DNA methylation in non-regenerative CNS. Surprisingly, however, axotomy led to widespread increases in DNA methylation in both regenerative regions of the CNS. Furthermore, in the two regenerative situations, these increases paradoxically encompassed multiple genes that increased in RNA expression after injury, analogous to what has been reported in mammalian stem cells and cancers [100]. Moreover, in optic nerve injury, these increases were clearly distinct from changes in 5hmC. These datasets provide evidence supporting the existence of an epigenetic switch underlying axonal regenerative potential in the vertebrate CNS and lay foundations for future work.

## Results

### Analysis of overall levels of DNA methylation demonstrated both developmental and injury-related differences between regenerative and non-regenerative CNS

To characterize developmental and injury-induced changes in DNA methylation (5mC) between axon-regenerative and non-regenerative regions of CNS genome-wide, WGBS was performed on spinal cord-injured (SCI) tadpole hindbrain (regenerative), optic nerve-crushed (ONC) frog eye (regenerative), SCI frog hindbrain (non-regenerative), and their respective controls at 15X genome coverage with three biological replicates each, as recommended [133].

**Fig. 1:**
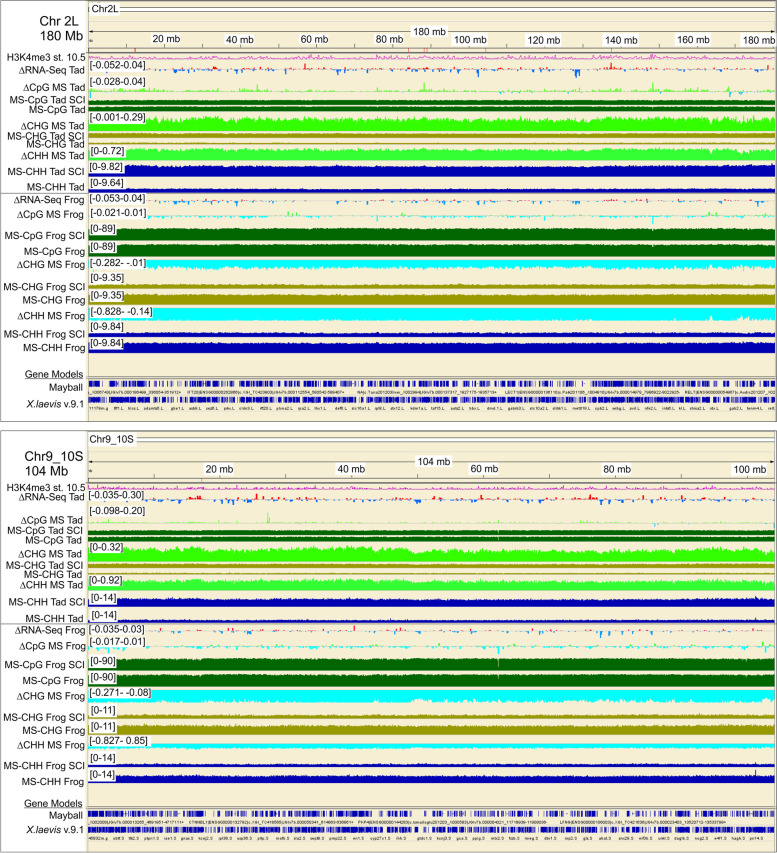
Chromosome-wide overview of injury-induced changes in DNA methylation for regenerative (tadpole) vs. non-regenerative (post-metamorphic frog) hindbrain after spinal cord injury (SCI) for two representative chromosomes (180 Mb of Chr 2L, top; 104 Mb of Chr 9_10S, bottom) as revealed by whole genome bisulfite sequencing (WGBS). Tracks for regenerative tadpole and non-regenerative frog hindbrain are grouped separately (Tad, top; Frog, bottom). For each chromosome, the vertical scales, which indicate the level of methylation (5mC) in each methylation context (CpG, dark green; CHG, olive green; CHH, navy blue), were group-autoscaled across tadpole and frog SCI and controls to facilitate comparisons between injury conditions (SCI vs. control) and developmental stage (tadpole vs. frog). Methylation differences between SCI and control (ΔCpG, ΔCHG, ΔCHH) indicate log_2_(SCI 5mC/control 5mC). The resulting increased (>0) and decreased methylation (<0) levels are shown in light green vs. blue, above and below the horizontal axes, respectively. Changes in RNA expression between SCI and control are also indicated (ΔRNA-Seq Tad and Frog; log_2_(SCI/control), with red and blue indicating increased and decreased expression, respectively [11]; note, RNA-Seq and WGBS were performed on RNA and DNA, respectively, isolated from the very same animals. H3K4me3 peaks at gastrulation (st. 10.5, [41]) and the locations of annotated genes (gene models: Mayball [21; 88; 89]; *X. laevis v. 9.1* [122]) are also indicated. For all three DNA methylation contexts (CpG, CHG, CHH), methylation levels increased between tadpole and frog stages pervasively across the entire chromosome, and SCI induced opposite, pervasive methylation responses (ΔCpG, ΔCHG, ΔCHH) in tadpole vs. frog [increased (light green) vs. decreased (light blue) methylation, respectively]. As illustrated in these two representative examples, similar patterns were seen for all chromosomes, with no overall differences between L and S homeologous chromosomes

**Fig. 2:**
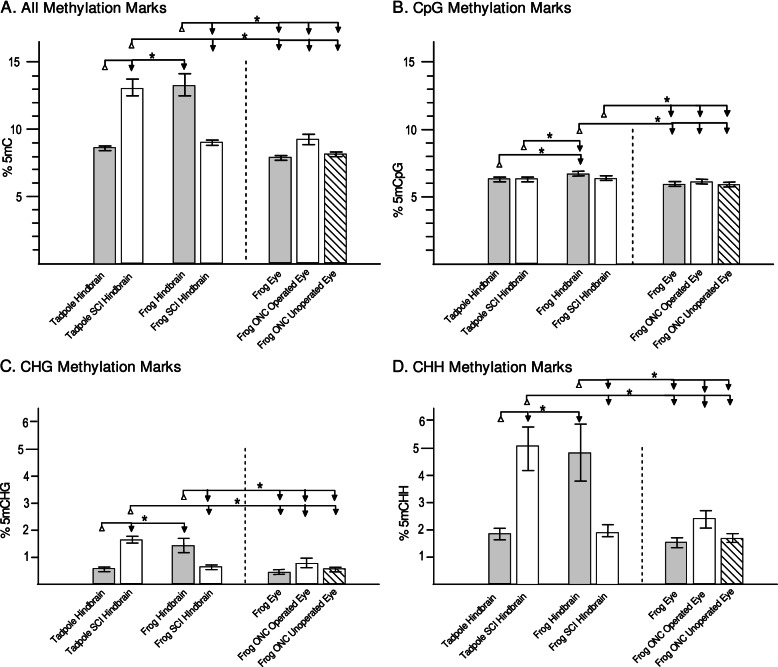
Quantitation of DNA methylation across the genome. Fraction of C’s exhibiting methylation marks in each context (**A**, % total 5mC; **B**, %CpG; **C**, %CHG; **D**, %CHH), as determined by WGBS, were averaged (±SE) across three biological replicates (5 pooled tadpole and frog hindbrains, 6 pooled frog eyes). One way ANOVA indicated that methylation differed significantly across all groups compared (P < 0.002). Results of *post hoc* comparisons are indicated by the brackets above (Fisher LSD; *, P < 0.05). See the text for further details concerning differences among conditions

We used premetamorphic tadpoles at NF stage 53 because our previous work empirically determined that tadpoles at this stage of development consistently and robustly regenerate damaged CNS axons with very high surgical survival rates (90%) and that thyroid hormone inhibits axon regeneration at this stage [36]. Moreover, hindbrains at NF stage 53 are well-developed, with a maximum number of neurons present before the onset of endogenous thyroid hormone secretion at NF stage 54 [65, 129]. The resultant WGBS data yielded high-resolution, quantitative information about both the level and the sequence-context (*i.e.*, CpG, CHH, or CHG) of the methylation. Viewed either at the whole chromosome level in Integrative Genomics Viewer (IGV; *e.g.*, illustrated in Fig. 1 for representative chromosomes Chr 2L and 9_10S for tadpole and frog hindbrain) or quantified across the genome for 5mC (Fig. 2, expressed as the average % total C ± SE), DNA of unoperated, non-regenerative post-metamorphic (frog) hindbrain exhibited significantly more methylation in all three contexts than did either of the two regenerative CNS regions, which in turn were more comparable to each other. For CpG methylation, the differences between regenerative and non-regenerative CNS, although small (~5%), were nonetheless statistically significant (P < 0.05, Fisher LSD *post hoc* test conducted after a one-way ANOVA (P = 0.0018) was performed on all samples). In contrast to the relatively modest differences seen for CpG methylation, differences for CHH and CHG methylation were markedly greater (Fig. 2), more than doubling between regenerative and non-regenerative CNS (2.5–3.2-fold; P < 0.05, Fisher LSD). Spinal cord injury (SCI) induced significant hypomethylation of CHH and CHG sites in non-regenerative frog hindbrain but the opposite in regenerative tadpole hindbrain (P < 0.05, Fisher LSD). Optic nerve injury induced analogous but more modest trends in CHH and CHG hypermethylation between the operated eye and controls than were seen for the regenerative tadpole SCI hindbrain (1.4–1.6-fold and 2.8-fold for ONC and SCI, respectively).

**Fig. 3:**
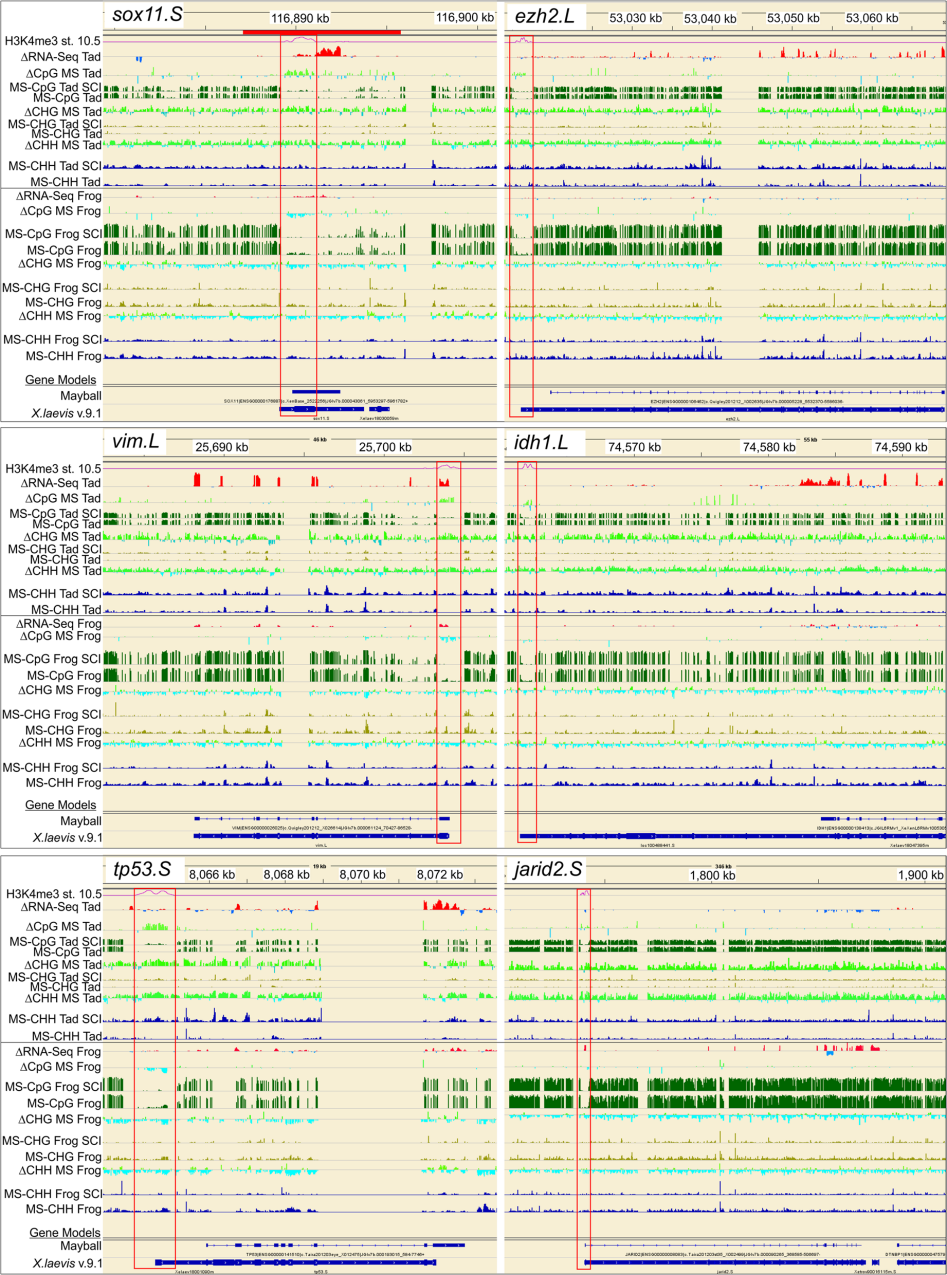
View of DNA methylation tracks (CpG, CHH, CHG) in hindbrain after SCI for representative genes known to be differentially expressed in successful vs. unsuccessful CNS axon regeneration [11]. Description of tracks and abbreviations are as in Fig. 1. Whereas changes in CHH and CHG methylation are pervasive across the genome, spanning both inter and intra genic regions, changes in CpG methylation are primarily confined to regions spanning the transcriptional start site (red boxes), where CpG methylation levels are generally lower than elsewhere.

For CpG methylation, the situation was more subtle than it was for non-CpG methylation. When quantified across the genome, injury-induced changes in CpG methylation were relatively minor compared to those seen for non-CpG methylation (< 5%, Fig. 2). Viewing local differences along the chromosomes in IGV revealed a pervasive preponderance of increased CpG methylation in regenerative CNS and decreased CpG methylation in non-regenerative CNS locally, analogous to what was seen for non-CpG methylation (*e.g.*, Fig. 1 for SCI and Fig. 10 for ONC; ΔCpG MS tracks, which graph the distribution of log_2_(fold-changes) along representative chromosomes, Chr 2L and 9_10S). Zooming in to the level of individual genes (Fig. 3) revealed that differences in CpG methylation were highly enriched in regions surrounding the transcription start sites (TSS), whereas injury-induced differences in non-CpG methylation (ΔCHH and ΔCHG MS) were more pervasive, affecting promoters, intragenic and intergenic regions alike. This is illustrated for six previously identified DESR genes (differentially expressed in successful but not unsuccessful CNS axon regeneration) [11] in regenerative tadpole (top of each panel) vs. non-regenerative frog hindbrain (bottom of each panel), between SCI and respective controls (Fig. 3). Data were comparable for ONC (not shown). Five of these genes (*sox11*, *ezh2, vim*, *idh1*, and *tp53*) increased and one (*jarid2*) decreased in successful regeneration (ΔRNA-Seq; red vs. blue for increased vs. decreased expression, respectively). Three of these genes also increase expression after axotomy under various regenerative conditions in mammals – *sox11* [49, 82, 123], *vim* [25, 84, 113], and *tp53* [50]. All six exhibited increased CpG methylation across the TSS (red boxes), as well as the more generally pervasive, increased non-CpG methylation across all regions, indicating that genes exhibiting SCI-induced hypermethylation in regenerative CNS included both up- and down-regulated genes. These genes were selected here to illustrate examples of the increased, promoter-associated CpG methylation seen in regenerative CNS. As seen in Fig. 1 for entire chromosomes, there were no systematic biases in DNA methylation between L and S homeologous chromosomes, and although only individual homeologs are illustrated for individual genes, patterns were generally similar with the other homeolog. An absence of methylation bias between homeologous chromosomes was also seen in *X. laevis* gastrula stage embryos [29]. As revealed in more detail below, subsequent analyses indicated that opposing patterns of methylation between regenerative and non-regenerative tissues were observed across a range of genes, regardless of their expression response to injury (up-, down-, and unchanging), and that not all differentially expressed genes exhibited promoter-associated changes in CpG methylation.

***Tissue- and injury-related changes in TSS-associated CpG methylation indicated their relationships with gene expression differed between regenerative and non-regenerative CNS***

**Fig. 4:**
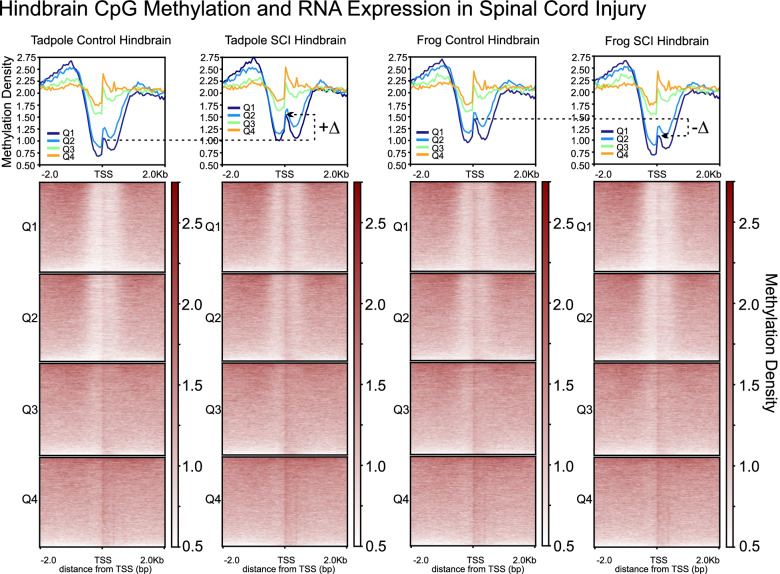
Degree of CpG methylation surrounding the transcriptional start site (TSS; ±2.0 kb) correlated with RNA expression for regenerative (tadpole) and non-regenerative (frog) hindbrain before and after SCI. **Top,** average level of CpG-methylation for different levels of gene expression (Q1 to Q4, representing the 25% most highly to least expressed genes, respectively from a total of 45,099 gene models in *X. laevis v.9.1* [122]). (+ or -) Δ, indicates the injury-induced changes for genes in Q1. x-axis units, distance from the predicted transcription start site (TSS) in kilobases (kb); y-axis units (Methylation Density), number of 5mCs in a 50 bp bin x 1 million/total number of Cs. **Bottom,** heatmaps of CpG methylation for each quartile, clustered from highest to lowest. The degree of CpG-methylation exhibited the expected negative correlation with RNA expression, but for the top two quartiles, it increased in regenerative hindbrain and decreased in non-regenerative hindbrain after SCI. The degree of CpG methylation is indicated by the intensity of the color, as indicated to the right of each heatmap (Methylation Density)

To visualize the relationships between promoter-region CpG methylation and gene expression across the entire genome more clearly, we generated heatmaps of CpG-methylation as a function of position relative to the TSS (± 2.0 kb) for genes within successive quartiles of RNA expression (Q1–Q4, ranked highest to lowest from a total of 45,099 gene models, *Xenopus laevis* v.9.1 [122]). Figure 4 shows the data for frog and tadpole SCI hindbrain and their respective controls (see Fig. 11 for ONC eye); bottom panels illustrate heatmaps, whereas top panels graph the averages for each quartile. These plots illustrate the well-known island-shore phenomenon of CpG methylation [24]. The TSS was surrounded by a region (± ~700 bp) of reduced CpG methylation compared to surrounding regions (CpG shore), and this depleted ‘well’ contained a narrower band (± ~250 bp) of relatively higher CpG methylation (CpG island). For each condition, the depth of these CpG ‘wells’ was inversely correlated with expression (*i.e.*, the deeper the well the greater the level of RNA expression), as has been described as typical for multiple systems [24]. For control hindbrain, genes falling within the two highest quartiles of RNA expression exhibited higher levels of CpG-methylation in non-regenerative frog than regenerative tadpole, consistent with the developmental increase illustrated earlier for CpG methylation along entire chromosomes (Fig. 1,2) and for multiple individual genes between these two stages (Fig. 3), whereas the lower two quartiles exhibited no discernible change. After SCI, the two higher quartiles exhibited opposite changes in CpG methylation across the TSS relative to controls between regenerative tadpole vs. non-regenerative frog [increased (labeled +Δ for Q1 between tadpole SCI hindbrain and age-matched control) vs. decreased (labeled -Δ for Q1 between frog SCI hindbrain and its control), respectively]. In the other regenerative tissue, optic nerve crush induced a similar, albeit smaller, increase in the operated eye relative to its controls as had occurred with tadpole SCI hindbrain (illustrated later, in Fig. 11A, to facilitate a direct comparison with hydroxymethylation in that tissue).

**Fig. 5:**
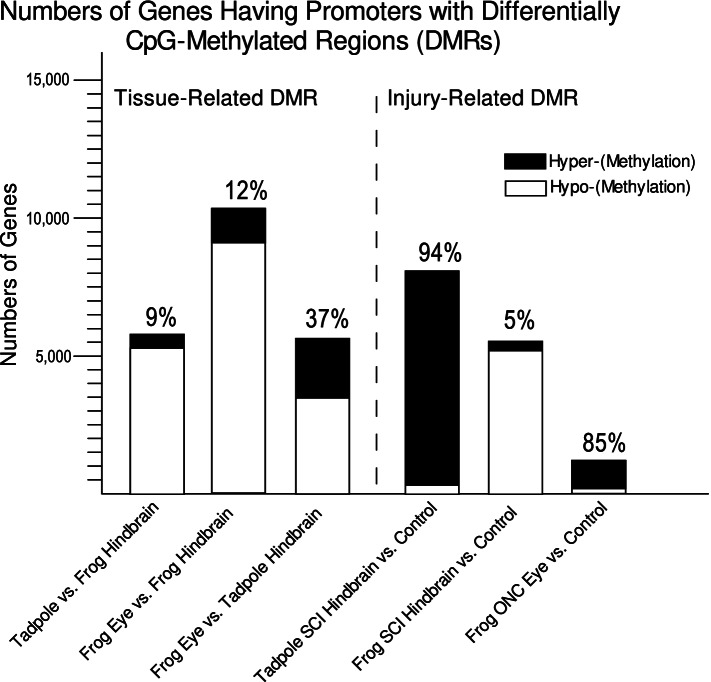
Numbers of genes having promoters (defined as 750 bp upstream to 250 bp downstream of the TSS) harboring differentially CpG-methylated regions (CpG-DMRs) for the various tissue- and injury-related comparisons. Bars indicate the total number of genes (from a total of 45,099 gene models in *X. laevis v.9.1* [122]) that harbored such CpG-DMRs. Numbers above each bar indicate the fraction of such genes with DMRs >0 between the first vs. the second listed condition (hyper-methylated CpG DMR; black). CpG DMRs between uninjured regenerative vs. non-regenerative tissues (*i.e.*, tadpole hindbrain and frog eye vs. frog hindbrain, respectively) were predominantly hypomethylated (CpG DMR < 0). With CNS injury (SCI or optic nerve crush (ONC)), CpG DMRs were predominantly hyper-methylated between injury vs. control conditions in regenerative CNS (*i.e.*, tadpole SCI hindbrain and frog ONC eye), and hypo-methylated in non-regenerative CNS (*i.e.*, frog SCI hindbrain)

To quantify the relative proportion of genes exhibiting increased vs. decreased CpG-methylation across the TSS, we identified differentially methylated regions (DMRs [32]) that fell within promoter regions (from 750 bp upstream to 250 bp downstream of the TSS), for both tissue-related and injury-related comparisons (Fig. 5). Comparing either of the two regenerative tissues (control tadpole hindbrain and eye) against the non-regenerative tissue generated substantially fewer hypermethylated DMRs in regenerative relative to non-regenerative CNS (9%, tadpole vs. frog hindbrain; 12%, frog eye vs. frog hindbrain) than hypomethylated DMRs (91%, tadpole vs. frog hindbrain; 88%, frog eye vs. frog hindbrain). This difference was consistent with the expectation based on mammalian studies that a decline in regenerative potential between tissues should be reflected in increased DNA methylation. Also consistent with this expectation, the balance between hypomethylated and hypermethylated DMRs between the two regenerative tissues in the absence of injury (tadpole hindbrain vs. frog eye) was more equitably distributed (37% hyper methylated DMRs; 63% hypo methylated). In sharp contrast, injury vastly favored hypermethylated over hypomethylated DMRs in the two regenerative CNS regions relative to their uninjured controls (94% and 85% of DMRs were hypermethylated relative to controls for tadpole SCI hindbrain and frog ONC eye, respectively). The opposite was the case in non-regenerative CNS (5% were hypermethylated relative to controls for frog SCI hindbrain vs. control).

**Fig. 6:**
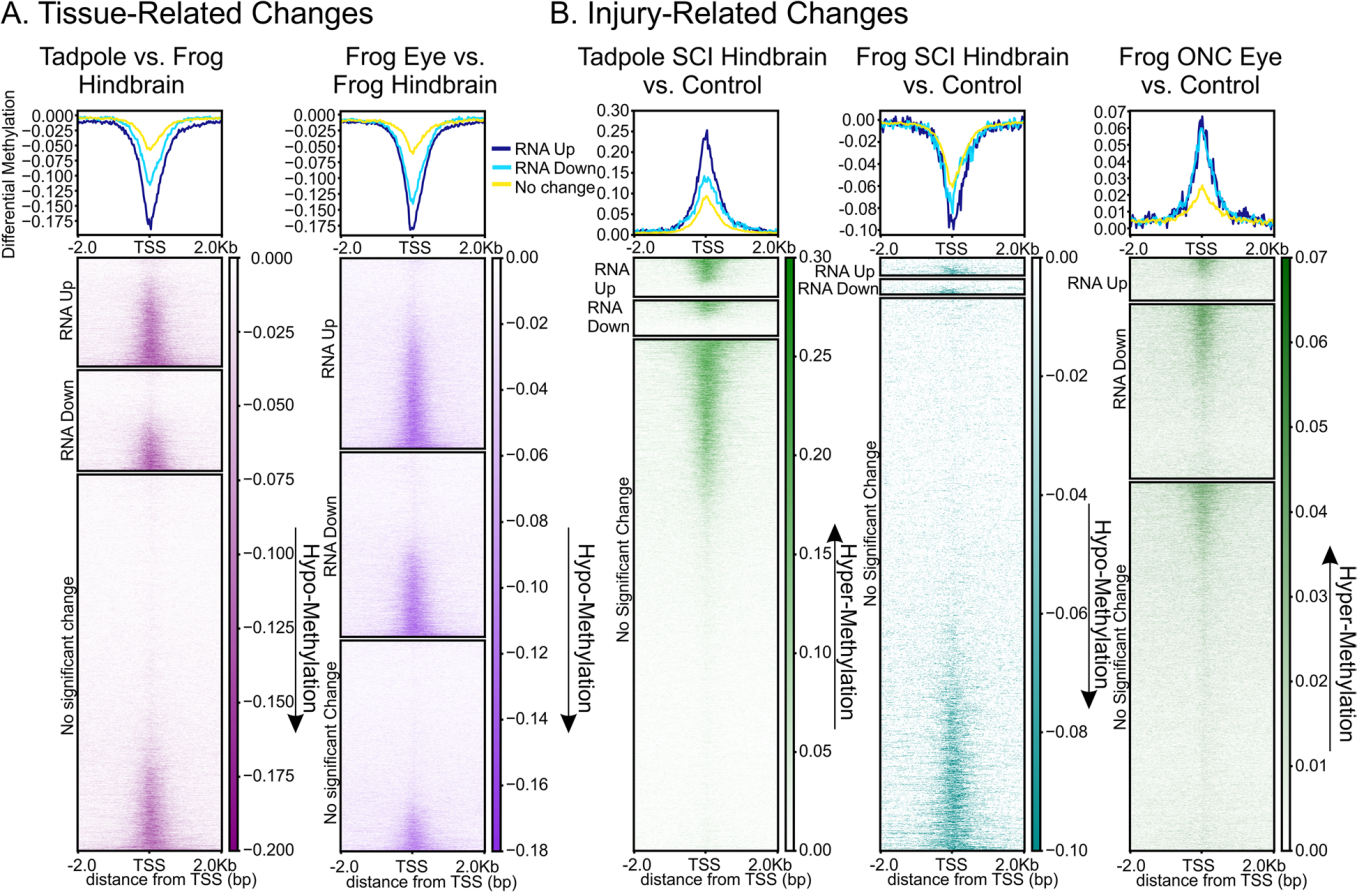
Tissue- and Injury-related changes in CpG methylation across the TSS (-2 kb – +2 kb) for all genes in the genome (N = 45,099 gene models), correlated with differential gene expression. Top and bottom panels illustrate average density and heatmaps of CpG methylation across the TSS for genes in each differential expression grouping. Whether a gene fell into the RNA Up, RNA Down, or no significant change category was determined by RNA-seq from RNA samples previously collected [11] from the same animals analyzed here by WGBS. Comparisons indicate changes between the first vs. second conditions (*e.g.*, negative differential methylation and RNA Up indicate hypo-methylation and significantly increased RNA expression, respectively, in the first vs. the second condition, etc.). Injury vs. control heatmaps for non-regenerative CNS (frog hindbrain) resembled those generated by comparisons between regenerative vs. non-regenerative CNS (tadpole and frog eye vs. frog hindbrain, respectively) and the opposite of what was seen with CNS injury in the two regenerative CNS regions (*i.e.*, tadpole SCI hindbrain and frog ONC eye). x-axis units, as in Fig. 4; y-axis units, Δ5mC within a 50 bp bin x 1 million/total number of Cs

To further visualize the relationship between injury-induced changes in CpG methylation across the TSS and differential RNA expression, we plotted differences in CpG methylation across the TSS (± 2.0 kb) separately for significantly (FDR < 0.05) up-regulated and down-regulated genes, and for those whose expression did not change significantly, for all genes (Fig. 6). These plots (top panel) confirmed that differences in CpG-methylation were enriched across the central CpG island. Consistent with all earlier comparisons and the canonical view of the relationship between CpG methylation at the TSS and differential gene expression [24], hypomethylation was favored in regenerative vs. non-regenerative CNS (Fig. 6A), was greater in genes exhibiting differential expression than in those that did not change between tissues, and favored increased over decreased RNA expression (top panels, dark blue vs. light blue lines; P < 0.0001 in a Pearson’s Χ^2^ 2x2 analysis of hyper- and hypomethylated DMRs vs. increased and decreased expression (Additional File 1), relative to the null hypothesis of no relationship between DMRs and RNA expression: tadpole vs. juvenile hindbrain, Χ^2^ (1, N = 2184 genes) = 36.58; frog eye vs. hindbrain, Χ^2^ (1, N = 5990) = 431.60]. A similar relationship, which was also comparable to what happens with non-regenerative CNS injury in mammals [13], was seen in non-regenerative frog hindbrain after SCI (Fig. 6B), although the relationship between DNA methylation and increased vs. decreased methylation was less pronounced than for tissue-related comparisons (P < 0.02, Χ^2^ (1, N = 536) = 5.30). Again, the opposite was seen in the two regenerative CNS regions after injury (Fig. 6B), where hypermethylation was favored. Although both regenerative tissues exhibited substantial numbers of up-regulated genes among hyper-methylated promoter regions, the bias between increased vs. decreased expression was not statistically significant [P = 0.23, tadpole SCI hindbrain vs. control, Χ^2^ (1, N = 1609) = 1.48; P = 0.72, frog ONC eye vs. control, Χ^2^ (1, N = 400) = 0.13]. This reduced bias argued that the increased injury-induced, TSS-associated CpG-methylation seen in regenerative CNS was more likely permissive than instructive for changes in gene expression.

**Fig. 7:**
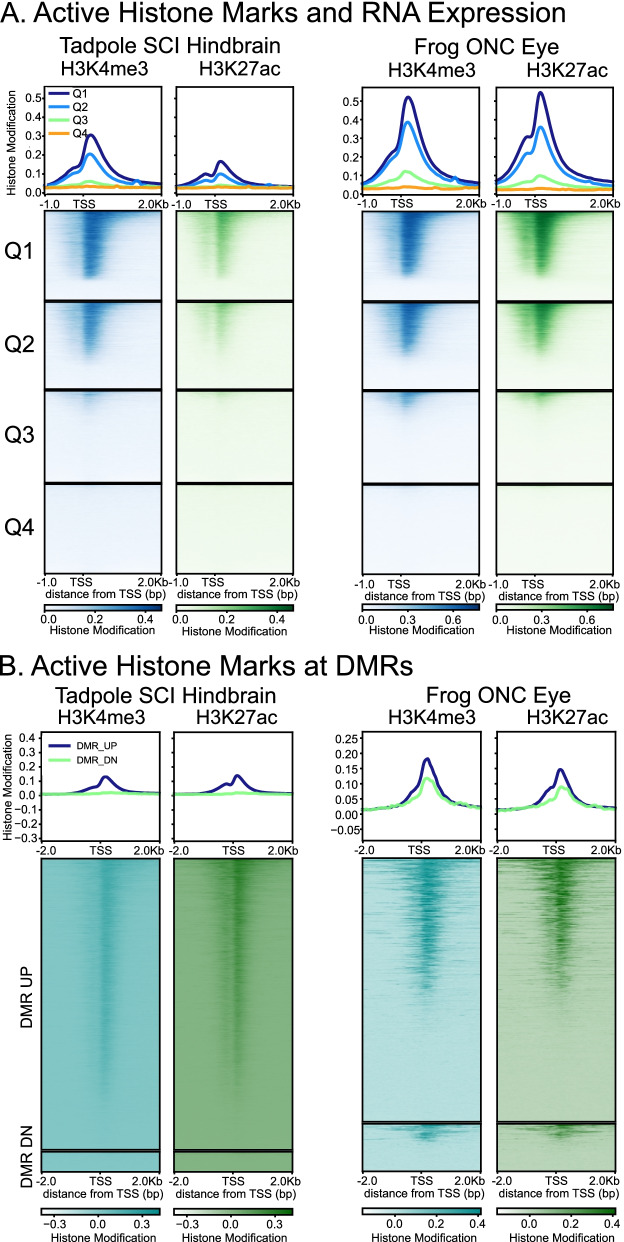
Heatmaps of active histone marks (H3K4me3 and H3K27ac) across the TSS, relative to quartiles of RNA expression (**A**) and Promoter CpG-DMR’s (**B**) in regenerative CNS tissues after SCI (left) and ONC (right). **A**, the presence of active marks declined with decreasing levels of RNA expression (Q1 to Q4, most to least). **B,** active histone marks were more highly represented among DMRs exhibiting increased methylation with injury (DMR Up) than among those exhibiting decreased methylation (DMR DN). See Fig. 5 for total numbers of DMR-bearing genes in each category. x-axis units as in Fig. 4; y-axis units, number of mapped reads in a 50 bp bin x 1 million/total number of mapped reads

Because RNA expression during successful CNS axon regeneration can be regulated post-transcriptionally as well as transcriptionally [1, 2, 85, 111], we performed ChIP-Seq for two histone modifications associated with active gene expression (H3K4me3 and H3K27ac [128]) in regenerative CNS (tadpole SCI hindbrain and frog ONC eye) to confirm whether injury-related, hyper-methylated CpG DMRs at the TSS were correlated with more active transcription (Fig. 7). First, we confirmed that the genome-wide density of these active marks across the TSS fell with decreasing RNA expression in regenerative CNS after injury (Fig. 7A; Q1>Q2>Q3>Q4), as expected from the typical behavior of these marks. Second, when we assayed the density of active histone marks across the TSS separately for hyper-methylated DMRs (DMR_UP) and hypomethylated DMRs (DMR_Down), we found that genes bearing hypermethylated DMRs were enriched for these marks, more so than were those with hypomethylated DMRs (Fig. 7B). As with earlier comparisons, this relationship was stronger for tadpole hindbrain than it was for frog eye (see Discussion).

### Gene ontologies of genes exhibiting injury-induced, increased TSS-associated CpG-methylation in regenerative tadpole hindbrain suggested unique functions

**Fig. 8:**
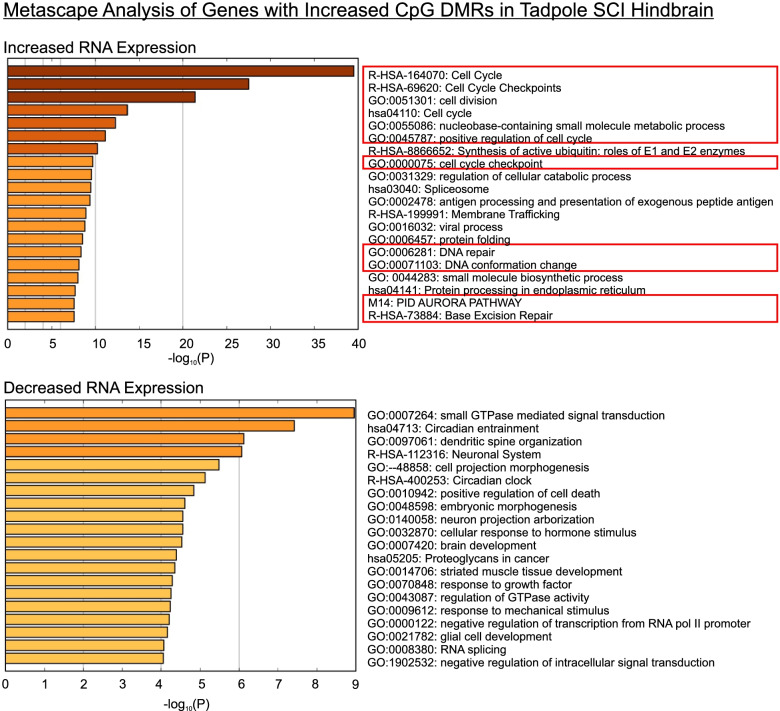
Gene Ontologies (Metascape [116]) for genes exhibiting increased CpG-DMRs after SCI in tadpole (CNS axon regenerative) hindbrain. The twenty highest ranking categories (*i.e.*, lowest probability (P) of arising by chance) are listed separately for up-regulated (top) and down-regulated (bottom) genes. Up-regulated genes were highly enriched for categories representing genes associated with DNA replication, repair, and covalent modification (red boxes). Injury-induced genes that decreased in expression had many genes associated with physiological functions and developmental functions (see text). Total numbers of genes in the top and bottom panels were 738 and 460, respectively

To determine whether genes exhibiting hypermethylated TSS DMRs in regenerative CNS were functionally related, we performed gene ontology analysis (Metascape [116]) on annotated genes that also saw significant changes in RNA expression (FDR < 0.05 [11]; Additional File 1). Because it had approximately eight times more such genes than frog ONC eye, the results were especially instructive for tadpole SCI hindbrain (Fig. 8). Injury-induced hypermethylated genes exhibiting increased RNA expression were enriched with very high probability (7 < -log_10_(P) < 40) for genes associated with a range of functions typically associated with dividing cells (*e.g.,* cell cycle, cell cycle checkpoints, cell division, positive regulation of cell cycle), and with DNA repair and covalent modifications (*e.g.*, nucleobase-containing small molecule metabolic process, DNA repair, DNA conformation change, AURORA PATHWAY, base excision repair). Such categories were consistent with the increased proliferation of macrophages and other myeloid cell types, reactive stem cells, and the enhanced epigenetic reprogramming that occurs in regenerative nervous systems [5, 27, 77, 117, 127]. Because of the fewer number of genes involved, hypermethylated genes exhibiting decreased RNA expression were enriched with generally lower probability (4 < -log_10_(P) < 9) for categories associated with physiological functions (*e.g.*, small GTPase mediated signal transduction, neuronal system, response to mechanical stimulus, negative regulation of intracellular signal transduction), neural development (*e.g.*, dendritic spine organization, cell projection morphogenesis, embryonic morphogenesis, neuron projection arborization, brain development, striated muscle tissue development, glial development), and cell death (positive regulation of cell death). Such categories were consistent with tadpole hindbrain transitioning away from its ongoing physiological functions and development after injury. This analysis helped strengthen the hypothesis that injury-induced CpG hypermethylation across the TSS was functionally related to genetic programs favoring recovery (see Discussion).

### Injury-induced changes in DNA hydroxymethylation were distinct from those of CpG methylation during optic axon regeneration

**Fig. 9:**
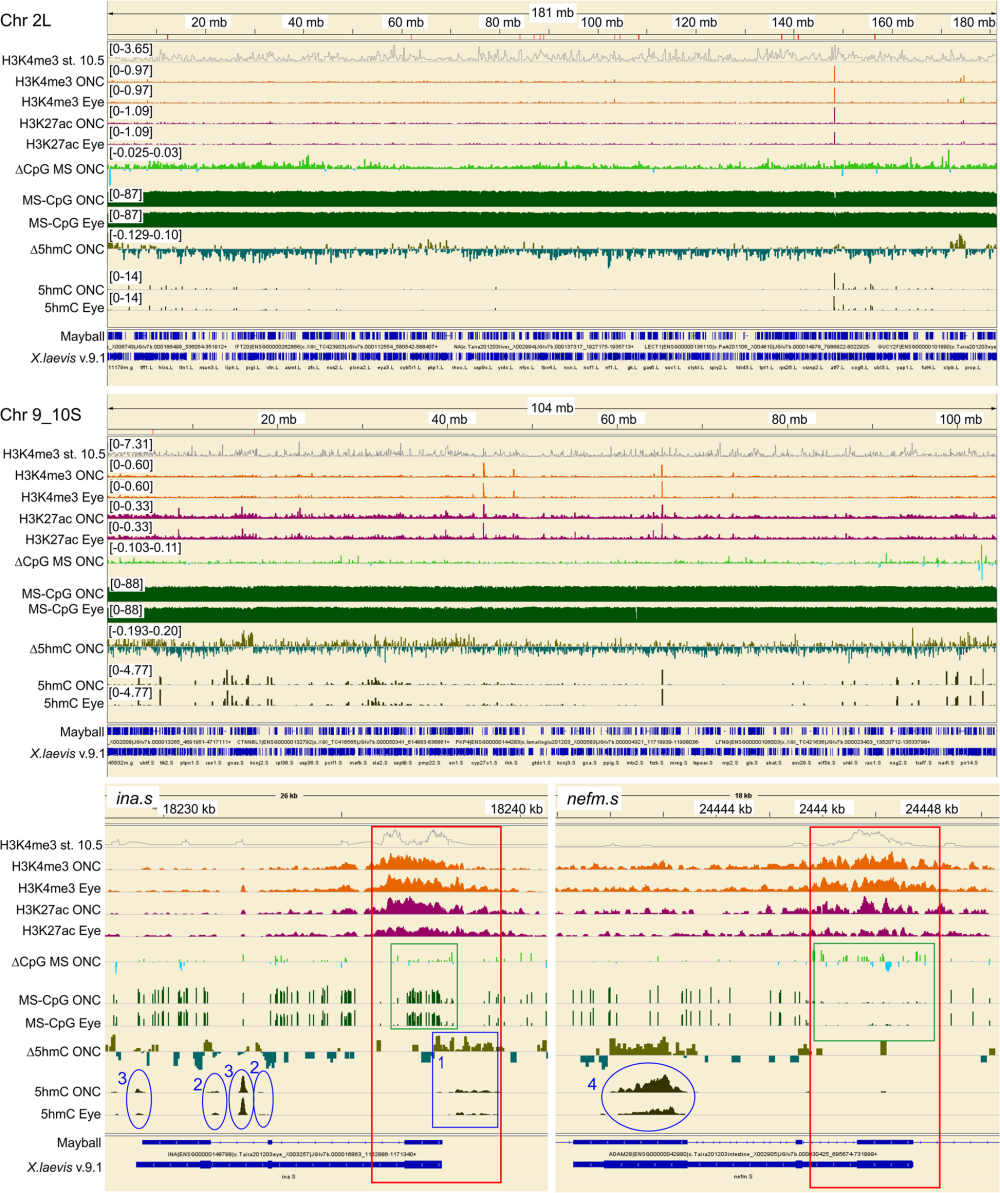
In optic nerve injury, changes in 5-hydroxymethylcytosine are distinct from those of CpG 5mC. Chr 2L and 9_10S: as was seen for SCI in tadpole hindbrain (Fig. 1, illustrated for the same two chromosomes), ONC-induced increased CpG methylation (ΔCpG MS ONC: log_2_(MS-CpG ONC/MS-CpG control eye)) pervasively across the entire chromosome (light green vs light blue for increased vs. decreased CpG MS, respectively). In contrast, 5hmC (Δ5hmC ONC) exhibited both increases and decreases spread across the chromosome. *ina.s* and *nefm.s*, two neuronal intermediate filament genes that increase with ONC in retinal ganglion cells, illustrate the complexity of 5-hydroxymethylation. Red boxes, regions encompassing the TSS marked by active histone marks (H3K4me3, H3K27ac). Green box, region encompassing the TSS, that exhibited increased CpG methylation (ΔCpG MS ONC, light green). 1, blue box, in *ina.s*, region near the TSS exhibiting increased 5hmC with injury (Δ5hmC ONC, olive green), which mostly flanked that marked for increased CpG 5mC. Note, *nefm.s*, exhibited little to no 5hmC marks in the corresponding region. Blue ellipses: **2**, 5hmC marks at the borders of intron 2 of *ina.s*. ***3***, 5hmC marking integrated adenoviral retroviral sequences in *ina.s*. **4**, 5hmC marking the template strand of a region of exon 3 of *nefm* that is highly enriched for repetitive glutamates. See text for more details

WGBS does not distinguish between 5mC and 5hmC. The latter is increasingly seen to be as important an epigenetic mark as 5mC in mammalian nerve injury and could conceivably explain the seemingly paradoxical correlation between increased DNA methylation and corresponding increases in gene expression seen in regenerative CNS after injury [62, 71, 75]. Thus, we sought to determine whether the increases in DNA methylation detected by WGBS in regenerative CNS might in fact be due to 5hmC. The likelihood of this initially seemed high, mainly because we had already observed differential expression of multiple genes associated with regulating DNA hydroxymethylation (*e.g.*, components of the Polycomb Repressive Complex such as *suz12* and *jarid2* [80], *idh1* [94, 118], *prmt1* [107]) among the DESR genes of our prior RNA-seq study [11]. To identify such changes, we performed 5hmC DNA immunoprecipitation sequencing (5hmC DIP-seq) on DNA from eye during optic nerve regeneration. We chose to use ONC eye rather than SCI hindbrain for this because there already exists extensive data on the expression and anatomical locations in retina of multiple genes undergoing differential expression in optic axon regeneration, which could aid in the interpretation of the results – *e.g.*, *ezh2, jarid2, suz12, prmt1* and *idh1* [11], *vim* [77], *nefm* [35, 130] and *ina* [35, 132]. Chromosome-wide views of CpG methylation in IGV (Fig. 9; MS-CpG, ΔCpG MS) confirmed that the eye underwent pervasive injury-induced increases in CpG methylation comparable to those seen in regenerative tadpole hindbrain (illustrated for the same chromosomes – Chr 2L & 9_10S). In contrast, increased 5hmC regions were continually interspersed among decreased 5hmC regions (Δ5hmC ONC), indicating that Δ5hmC exhibited far more variation among individual genes than was the case for Δ5mC.

Higher resolution views of individual genes better illustrate examples of the range of gene-specific variation in 5hmC induced during regeneration. For example, the bottom of Fig. 9 illustrates 5mC WGBS and 5hmC DIP marks for two well-studied neuronal intermediate filament genes, *ina* and *nefm.* These genes are *Xenopus* orthologs of human alpha-internexin and the middle neurofilament protein, respectively, and their expressions both increase in retinal ganglion cells during optic axon regeneration [35, 130–132]. *Nefm* especially, and *ina* to a lesser degree, exhibited increased CpG methylation across promoter regions encompassing the TSS, which are also marked by ChIP-seq marks for active promoters and enhancers (H3K4me3 and H3K27ac; red box). These marks extended across the first exon and into the first intron, consistent with active enhancers of neurofilament genes extending into these regions [106]. Within this region, the 5hmC marks mostly flanked the regions exhibiting ΔCpG WGBS marks for *ina.s* (blue box #1) and were essentially absent for *nefm.s. Ina.s* had four additional zones of extensive 5hmC marks (blue ellipses, two labeled #2 & two labeled #3). Two (#2) marked the borders of intron 2, consistent with reports of hydroxymethylation occurring at the intron borders of multiple genes from insect to mammal [22, 28, 124]. The remaining two (#3) marked the locations of integrated adenoviral sequences in *Xenopus laevis*. This sequence (CCTACTATAC CTGCTATCCC ACAGTCACAC TTCCCTTCCC AGAGAC) represented ~10% of all the 5hmC immunoprecipitated sequences. A blast search of the entire NCBI database returned forty *Xenopus laevis* genes containing this sequence, including *ina.s*, plus several adenoviral sequences. The right-most 5hmC-marked region contained two copies of this sequence, and the left-most contained one. Methylation and hydroxymethylation of integrated retroviral sequences are an important silencing mechanism [44], suggesting that these marks function similarly. *Nefm.s* had an additional zone of high 5hmC (#4) within the last exon of *nefm.* This zone contains more than a hundred highly repetitive glutamates [34]. Because glutamate codons are GAA and GAG, the 5hmC must necessarily mark the complementary template strand sequences, which comprise repetitive TTCs and CTCs, respectively. Because these sequences are generally devoid of paired CpGs and rich in potential non-CpG sites, and because this extensive hydroxymethylation increased markedly with RNA expression, it seems reasonable to conclude that the increased non-CpG methylation seen in regeneration serves as a template for this hydroxymethylation, which in turn helps facilitate transcription of this highly repetitive sequence.

**Fig. 10:**
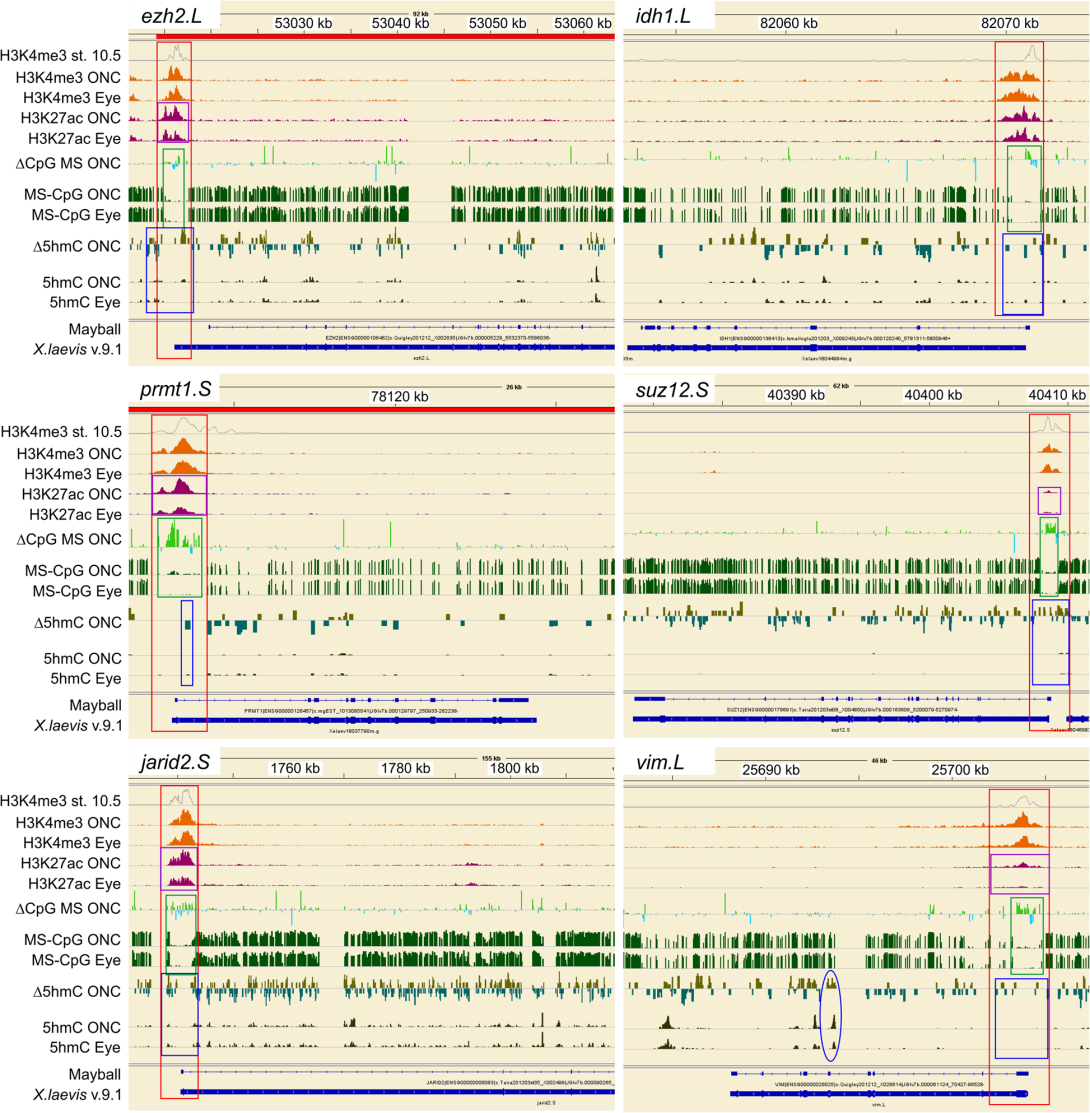
Additional examples of 5hmC vs CpG 5mC for representative genes changing in expression during ONC. Track labels, as well as red, green, and blue boxes, are as in Fig. 10. Magenta boxes, examples of increased H3K27ac marks induced by ONC. All genes exhibited the same behavior with respect to CpG as illustrated previously for *ezh2.L, vim.L, idh1.L* and *jarid2.S* in tadpole hindbrain after SCI (Fig. 3). Changes in 5hmC (Δ5hmC ONC: olive green, up; blue green, down) across the TSS were generally not congruent with those of CpG 5mC marks (blue boxes vs. green boxes, respectively). Blue ellipse, *vim.L*, an integrated adenoviral sequence marked by 5hmC

Further examples of 5hmC marks are illustrated for six additional genes that are differentially expressed in successful optic nerve regeneration (Fig. 10) – four represent up-regulated genes (*ezh2, idh1, prmt1, vim*) and two represent down-regulated genes (*jarid2*, *suz12*). These genes also exhibit differential expression in different retinal cell types. *Prmt1* increases in all retinal layers, whereas *ezh2, idh1*, *suz12,* and *jarid*i2 are differentially expressed in retinal ganglion cells [11]. *Vim* expression is newly induced in reactive Müller radial glia [77], which in vertebrates are stem cells [95, 99]. All six genes exhibited increased CpG methylation across the TSS (green box), which had been illustrated previously in regenerative tadpole SCI hindbrain for four of these genes in regenerative tadpole SCI hindbrain (Fig. 3; *ezh2*, *idh1*, *vim*, and *jarid2*). Again, the 5hmC marks in all these genes were generally distinct from WGBS CpG marks, consistent with what has been reported in mammals [75]. Whereas the largest injury-induced differential CpG WGBS marks were found in the region encompassing the TSS (red box encompassing the ChIP-seq histone marks for active promoters and enhancers), injury-induced differential 5hmC marks (Δ5hmC ONC) were liberally spread across the intragenic regions of these genes and exhibited both increases (olive green) and decreases (dark green). In addition, the ellipse in *vim*.*L* marks the location of a second integrated adenoviral sequence (GGGAAGGGAG TGTGACTGTG GGATAGCAGG TATAGTAGGG AGAGATGGTG), which like the first adenoviral sequence comprised ~10% of the 5hmC-immunoprecipitated sequences. Its blast search found 96 examples of this sequence distributed across both *Xenopus laevis* and *Xenopus tropicalis* genes, in addition to several adenoviral sequences.

**Fig. 11:**
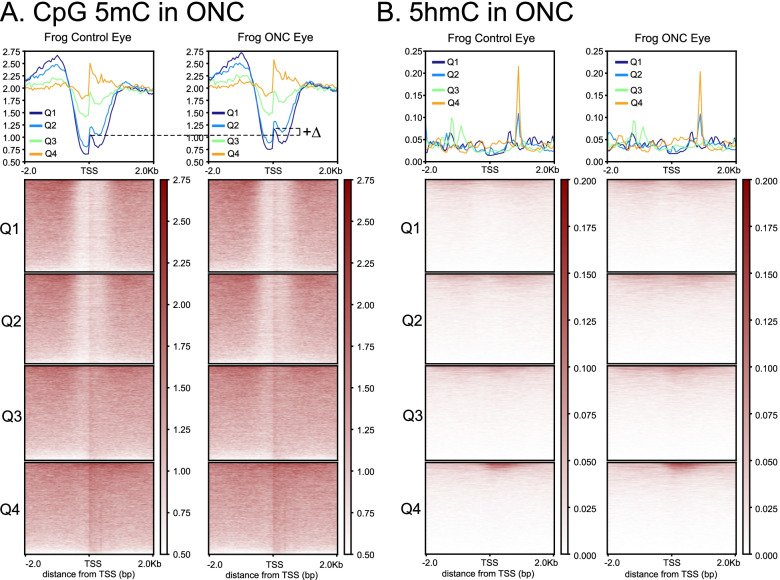
Distributions of CpG 5mC surrounding the transcriptional start site (TSS; ± 2.0 kb) to ONC differed markedly from that of 5hmC. **A,** Although less pronounced than in tadpole SCI, the density of CpG DMRs across the TSS follows the same pattern as for tadpole SCI, with CpG 5mC DMRs increasing for the top two quartiles of RNA expression with injury. Units and labels are as in Fig. 4. **B,** In contrast, 5hmC showed no such response, and overall was markedly different from the pattern of CpG 5mC (see text for details). Units and labels are as in Fig. 7

To further confirm the distinct natures of Δ5hmC from ΔCpG methylation, we compared the density of the two across the TSS (± 2.0 kb) during ONC, for decreasing quartiles of RNA-expression for all genes (Fig. 11A), as had been done for hindbrain (Fig. 4). The injury-induced increase in CpG methylation between operated and control eyes (Fig. 11A: *e.g.*, Δ+ for Q1, top panels) was analogous, albeit less pronounced, than that illustrated previously for regenerative tadpole SCI hindbrain (Fig. 4). In contrast, the 5hmC plots (Fig. 11B) were quite different from the CpG WGBS plots. Instead of the shores and islands that were characteristic of CpG methylation, the distributions of 5hmC marks were essentially flat across the TSS, except for two sharp spikes, which appeared approximately 1 kb intragenic from the TSS for the two least-expressing quartiles (Q3 & Q4). The absence of this spike from the upper two quartiles suggested it is an inhibitory feature. Thus, despite the numerous injury-induced differences seen for 5hmC among individual genes, there were no consistent genome-wide, injury-induced differences that correlated with RNA-expression for 5hmC across promoter regions. Thus, collectively, these data indicated that the differential TSS CpG methylation seen with CNS injury was a separate and distinct feature from injury-induced changes in 5hmC, which were both more broadly distributed across and unique for each gene.

## Discussion

To identify differences in DNA methylation between successful and unsuccessful CNS axon regeneration, we mapped DNA methylation by bisulfite WGBS at high resolution during the peak period of regenerative axon outgrowth in frog eye and tadpole hindbrain (regenerative) vs. frog hindbrain (non-regenerative) during ONC and SCI, respectively. To correlate changes in DNA methylation with gene expression, the density of these marks was compared with RNA-expression using RNA-seq data collected from the same animals [11] and with the distribution of histone markers for active gene expression (H3K4me3 and H3K27ac) by ChIP-seq. When viewed between separate tissues with different regenerative capacity (frog hindbrain vs. eye) and between the same tissue collected before and after regenerative capacity is lost during development (tadpole vs. frog hindbrain), DNA methylation behaved largely as anticipated from mammalian studies [14, 24, 40, 67, 72] – namely, baseline levels of DNA methylation were higher in non-regenerative than in regenerative CNS and differences in CpG methylation in promoter regions were inversely correlated with differential gene expression. Also, as in mammals [13, 46], axotomy of non-regenerative CNS (frog hindbrain after SCI) induced lower overall levels of DNA methylation, which for CpG methylation at promoters was negatively correlated with gene expression. In sharp contrast, axotomy of regenerative CNS (tadpole hindbrain and frog eye after SCI and ONC, respectively) induced overall increases in DNA methylation. Particularly in the case of CpG methylation in promoter regions, this increased methylation was paradoxically associated with increased gene activity. Multiple studies in regeneration-competent animals (*e.g.*, zebrafish and *Xenopus*) and in mammals have indicated that maintaining and even increasing chromatin accessibility are important for eliciting regenerative potential [13, 51, 117, 119]. Thus, these increases likely represented an opening-up of the genome to make it more accessible to the transcription factors that regulate the axotomy-induced changes in gene expression that orchestrate a successful recovery from CNS injury. This seemingly paradoxical relationship between injury-induced increased DNA methylation and gene activation in regenerative *Xenopus* CNS is novel for axon regeneration and could provide a foundation for future biochemical studies aimed at understanding molecular mechanisms underlying successful CNS axon regeneration.

Changes in non-CpG methylation (CHH and CHG) were particularly striking. They occurred pervasively across the genome, both in development and with injury. DNA methylation in this context was also significantly higher in non-regenerative CNS than in either regenerative CNS region, and after axotomy, it decreased in non-regenerative CNS and increased in regenerative CNS. These changes were spread evenly across the genome, affecting promoter regions, intragenic, and intergenic regions alike. Phylogenetically, non-CpG DNA methylation first emerges in vertebrates, where it is generally enriched in sets of developmental genes as they become transcriptionally repressed in adults [23]. Mammalian brains exhibit high degrees of non-CpG methylation, wherein it plays a critical role in cognitive brain function. There, it increases, as it did in *Xenopus* hindbrain development, during later developmental stages at a time generally associated with increased synaptogenesis and dendritic arborization [23, 40, 48, 67]. Because in mammalian brain, non-CpG methylated sites bind the transcriptional repressor, MeCP2, and are negatively correlated with gene expression, they, like methylated CpGs in promoter regions, are generally considered inhibitory for gene expression [23, 40, 48, 86]. However, the abundance of non-CpG methylation in pluripotent stem cells suggests its relationship with gene expression may be more complex, especially earlier in development [48, 68, 134]. In mammals, non-CpG methylation is performed by the same enzymes that catalyze *de novo* CpG methylation, DNMT3a and DNMT3b [86, 134]. Whereas DNMT3a preferentially methylates CAC sequences, which are the primary target of MeCp2, DNMT3b preferentially methylates CAG sequences, which are not targeted by MeCp2. Interestingly, DNMT3a and b are preferentially expressed in neurons and stem cells, respectively, suggesting non-CpG methylation in mammals may be inhibitory in neurons but not in stem cells [63]. Because *Xenopus* expresses only a single DNMT3, the non-CpG methylation we observed in *Xenopus* could thus represent a melding of the two separate contexts seen in mammals. If so, it raises the intriguing possibility that the increased non-CpG methylation seen in regenerative CNS in *Xenopus* could be functionally related to what happens in activated stem cells in mammals. Also, because many of the regions exhibiting injury-induced increases in 5hmC lack CpG dinucleotides, increased levels of non-CpG methylation seen in successful axon regeneration may help provide the needed substrate for increased 5hmC for some genes.

For much of this study, we concentrated on what happened with CpG methylation in promoter regions, primarily because the relationship between it and gene activation is better understood [24]. Because increased gene activation is usually associated with reduced CpG methylation at promoters, we initially anticipated that regenerative CNS would undergo more dramatic reductions in CpG methylation across promoter regions after axotomy than would non-regenerative CNS, but instead observed the opposite. Interestingly, some earlier studies of nerve regeneration in rodents have hinted that a similar paradoxical relationship might exist between increased DNA methylation and activation of pro-regenerative genes in nerve regeneration, leading some authors to conclude that the relationship between DNA methylation and gene expression in nerve injury is somewhat ambiguous [13]. For example, in spinal cord, stimulating folate-metabolic pathways, which activate DNMTs, not only inhibits the DNA demethylation typically observed in mammalian SCI, but paradoxically, also increases axon regenerative potential [46]. And, in dorsal root ganglionic (DRG) neurons, which successfully regenerate peripheral axons, sciatic nerve injury increases DNMT3b expression as these neurons newly activate growth-associated programs of gene expression [87]. Such paradoxical observations were initially explained by invoking the ability of DNMTs to recruit histone deacetylases to promoters to activate genes. After it was realized that activating folate-dependent metabolic pathways also increases levels of 5hmC, and that 5hmC is both an intermediate step along the pathway to DNA demethylation and a stable DNA modification in and of itself, investigators focused on hydroxymethylation as a possible resolution to these paradoxical observations [6, 55, 78, 83]. 5hmC is enriched in many actively expressed genes and varies developmentally among cell types [67, 72]. In mammalian genes, 5hmC marks are widely distributed across intragenic sites, often negatively correlated with repressive histone marks (H3K9me3 and H3K27me3), and positively correlated with markers for poised enhancers [72], plus it is an intermediate step along the pathway to demethylating 5mC. Hence, it was thought that hydroxymethylation could reasonably explain why stimulating folate pathways can both increase DNA methylation and activate genes promoting axon regeneration. Indeed, such a functional relationship was demonstrated in studies showing that injuring the regenerative nerve branch of DRG neurons leading to the PNS, but not the non-regenerative branch leading to the CNS, selectively increases 5hmC and TET activity, the enzyme that converts 5mC to 5hmC, in DRG neurons as they activate regeneration-associated genes. This increased 5hmC occurs primarily within gene bodies but also sometimes occurs ~1kb upstream of the TSS [71], and it targets CpG and non-CpG sites alike [73]. In mammalian PNS, which can regenerate its axons, axotomy stimulates DNA demethylation via upregulation of TET3, which forms the 5hmC intermediary from 5mC, preceding DNA demethylation, and inhibition of this enzyme inhibits axonal regrowth. Also, in mammalian CNS, TET1 knockdown leads to the reversal of the otherwise pro-regenerative effects of PTEN knockout in mice [8, 125, 126]. Thus, in mammalian nerve injury studies, several converging lines of evidence support a functional role for DNA hydroxymethylation in promoting nerve regeneration. Because both 5mC and 5hmC are injury-induced and seldom overlap in mammalian nerve injury studies, it is now considered important to distinguish between the two states [75].

Our own results in *Xenopus* optic nerve regeneration contribute further evidence that 5hmC is an important epigenetic component of successful CNS axon regeneration. Our earlier RNA-seq study had already found that expression of two genes associated with DNA hydroxymethylation – *idh1* and *prmrt1* – are selectively up-regulated in the two regenerative but not the non-regenerative CNS region [11]. IDH1 metabolically up-regulates TET enzymatic activity, promoting conversion of 5mC to 5hmC [94, 118], and PRMT1 associates with hydroxymethylated sites along DNA, where it methylates histones to regulate transcription [107]. PRMT1 also methylates the RNA-binding protein, hnRNP K, which is required for successful optic axon regeneration [18, 19, 70, 112]. In *Xenopus* retina, at the peak period of optic axon regeneration, *in situ* hybridization has previously demonstrated that both genes are upregulated in retinal ganglion cells [11], which provide the source of regenerating optic axons. Consistent with reports in mammals, sites undergoing injury-induced changes in 5hmC during *Xenopus* optic axon regeneration were more heterogeneous than those undergoing changes in CpG and non-CpG methylation. But most importantly for the current study, they were clearly distinct from the increases seen in CpG methylation in promoter regions, which is also generally the case in mammals [75].

The complexity of the 5hmC response seen in *Xenopus* optic nerve regeneration suggests that its functions extend beyond simply regulating transcription. For example, injury-induced changes in 5hmC marked some exon-intron boundaries (*e.g.*, *ina.s*), as well as the coding regions of exons of up-regulated mRNAs (*e.g.*, *nefm*). In the case of *nefm,* which is a gene that is exclusively expressed and highly upregulated in retinal ganglion cells during optic axon regeneration [1, 35], these increases covered a region of the last exon that is rich in highly repetitive glutamates [34] (Fig. 9, bottom right). They were also observed over a comparable, glutamate-rich region of *nefl,* another neurofilament gene (not illustrated). Because only the template strand of these regions contains C’s, 5hmC seems likely to somehow facilitate the reading of these highly repetitive sequences by RNA pol II. Even when 5hmC marks were found near the TSS, they typically flanked regions of CpG methylation. Such flanking 5hmCs are also seen in mammals, where they are thought to help limit incursions of CpG methylation into island-shores from surrounding regions [28]. The complexity of the 5hmC response clearly calls for more detailed studies, conducted on a gene-by-gene basis. Nonetheless, these observations support the conclusion of the current study, that the unusual increase seen in CpG methylation across promoter regions could not reasonably be attributed to increased hydroxymethylation. Thus, we conclude that the novel, injury-induced increase in promoter-associated CpG methylation seen in the two regenerative CNS regions likely represents an injury response that is characteristic of successful CNS axon regeneration.

Injury-induced changes in the expression of several enzymes that catalyze CpG methylation also support this conclusion [11]. Because it lacks an ortholog to *dnmt3b*, *dnmt3a* is fully responsible for *de novo* DNA methylation in *Xenopus* [59]. Consistent with the demethylation seen in the non-regenerative frog hindbrain after SCI, *dnmt3a* expression decreased significantly there by 37% after SCI (FDR < 0.003), whereas it changed only insignificantly and remained strongly expressed after axotomy in regenerative CNS (FPKM >40 and >6, in tadpole SCI hindbrain and frog ONC eye, respectively). A possible boost to DNMT activity in regenerative CNS after injury is instead provided by increased expression of *ezh2*, which helps target DNMT to promoters [69, 121]. In both ONC eye and tadpole SCI hindbrain, its expression increases significantly by ~50% (FDR < 0.002), and in ONC eye, *in situ* hybridization has localized this increase to retinal ganglion cells [11]. In contrast, in non-regenerative CNS, *ezh2* expression decreases significantly after SCI in the non-regenerative frog hindbrain (~50%, FDR < 0.003). Finally, a gene encoding an enzyme that directly converts 5mC to 5C, the cytidine deaminase *apobec3a* [17], decreases in expression significantly (FDR < 0.03) by 30–40% in both regions of regenerative CNS, whereas it increases slightly by 20% (FDR > 0.3; although P = 0.04) in non-regenerative CNS [11]. Thus, these changes working collectively together should favor increased methylation over de-methylation in regenerative CNS and vice versa in non-regenerative CNS.

Although novel for neural regeneration, our finding that increased promoter-related CpG methylation was associated with increased gene activation in successful CNS axon regeneration nonetheless has precedents in studies now emerging from cancers, stem cells, and development [100]. For example, in early malignancy, reactivation of the Wilms tumor 1 gene (*wt1*) is coupled with hypermethylation of its promoter, and echoing our own findings in *Xenopus*, this increase is due to increased 5mC and not 5hmC [39]. Also, in multiple tumor cell lines, the transcription factor *ebf3*, which is essential for metastasis, exhibits paradoxical hyper-methylation in its promoter during gene activation [20; 97]. Interestingly, *ebf3* is preferentially upregulated in successful *Xenopus* CNS axon regeneration and not in unsuccessful regeneration [11]. A general theme emerging from these mammalian studies is that many of the genes exhibiting this paradoxical methylation are involved in regulating cell division, cell cycle, and cell migration. Indeed, this theme was reiterated in the functional ontologies of genes undergoing injury-induced, increased promoter-associated CpG DMRs and increased RNA expression in regenerative tadpole SCI hindbrain (Fig. 8). These physiological processes are activated in axotomized neurons and their surrounding support cells. For example, successful CNS regeneration in *Xenopus* is supported by activation of proliferative macrophages and radial glia [27, 77, 127], and many genes activated in cancer metastasis are also involved in axon outgrowth (*e.g.*, [7]). Because injury-induced, hypermethylated DMRs included up-regulated genes that are relatively specific for neurons and for radial glia, such as *nemf.s* and *vim.L*, respectively [35, 77, 103, 104], the response in regenerative CNS seems likely to encompass multiple cell types. Because the increased promoter-region CpG methylation was also seen in down-regulated genes, it seems entirely possible that the affected up-regulated and down-regulated genes were expressed in separate cell types, with one behaving paradoxically and the other more conventionally. Thus, our results could in part be explained by injury-induced, shifting populations of cells differing between regenerative and non-regenerative CNS. However, hypermethylated promoter-associated CpG DMRs occurred in at least some genes that are entirely neuronal (*e.g.*, *nefm.s*), and increased non-CpG methylation was pervasive across the genome. Thus, at this time, the more parsimonious interpretation seems to be that increased DNA methylation is permissive rather than instructive, helping establish an appropriate environment for transcription factors that regulate increased and decreased gene expression, comparable to what is believed to occur in mammalian cancers and stem cells [100]. Future single-cell studies are needed to fully resolve this issue.

The precise mechanisms linking paradoxically increased CpG methylation across promoters with gene activation in mammals are still under investigation. Accumulating evidence supports several possibilities [100]. These include commonly understood mechanisms such as increased or decreased association with activating or repressive transcription factors and histone modifications, as well as novel mechanisms such as increased association of genes with the nuclear lamina to promote an open chromatin configuration [42, 100, 108]. The injury-induced modifications we observed in axon-regenerative CNS are thus likely to involve one or more of these mechanisms. Mechanisms that promote chromatin accessibility seem especially relevant since ATAC-seq studies have now demonstrated, both in cortical injury in mammals and in tail regeneration in *Xenopus* tadpole, that chromatin accessibility is important for eliciting regenerative potential [51, 119].

Our finding distinct DNA methylation responses in regenerative vs. non-regenerative CNS also fits nicely with an emerging story linking thyroid hormone exposure during metamorphosis with epigenetic changes underlying regional differences in global gene expression, as well as regenerative capacity, in the vertebrate CNS. In *Xenopus,* increased exposure to thyroid hormone during metamorphosis is directly responsible for the loss of axon-regenerative potential seen with SCI in hindbrain [36], yet exposure to the same increases has little effect on the axon-regenerative capacity of frog retinal ganglion cells [10, 33, 101, 105]. Proper control of DNA methylation is indeed essential for normal development in *Xenopus*, and exposure to thyroid hormone directly increases expression of *dnmt3a* in tadpoles [57, 59]. In *Xenopus* thalamus and hypothalamus, DNA methylation begins to increase at the onset of metamorphosis (st. 55), but then reverses after metamorphic climax, leaving it substantially de-methylated in froglets compared to tadpoles at metamorphic climax (st. 60) [58]. This demethylation responds directly to thyroid hormone [91] and is accompanied by increased levels of TET expression and DNA hydroxymethylation in thalamus and hypothalamus but not in hindbrain and spinal cord [58]. Thus, the expectation is that levels of DNA methylation should remain high in CNS regions outside the thalamus and hypothalamus after metamorphosis. Our finding higher levels of DNA methylation in the hindbrains of post-metamorphic frog than in premetamorphic (st. 53) tadpole hindbrain is fully consistent with these findings. Although we did not assay DNA methylation across metamorphosis in the eye, it seems reasonable that DNA methylation levels there would remain lower after metamorphosis than in hindbrain, because retina, thalamus, and hypothalamus are embryologically all derived from the same regions of the embryonic neural plate and neural tube as each other [26, 45, 102]. Notably, thyroid hormone also directly stimulates *dnmt3a* expression while triggering the loss of regenerative capacity that occurs during late fetal development in mammals [4, 60]. Because both post-metamorphic frog hindbrain and mammalian CNS are non-regenerative, the similarities in the injury-induced DNA-methylation response between them are likely to have similar, phylogenetically conserved functional consequences. Thus, the seemingly paradoxical folate-response that seems to promote both DNA methylation and neural regeneration in mammals [46] may represent the vestiges of a more vigorous pro-regenerative response to neural injury inherited from their anamniote ancestors.

## Conclusions

We conclude that the axotomy-induced changes in DNA methylation in regenerative CNS that we report here provide strong evidence for a novel epigenetic state favoring successful over unsuccessful CNS axon regeneration. While much remains to be discovered about this phenomenon, the extensive datasets described here can provide a firm foundation for future studies of the molecular and cellular mechanisms involved and their implications for potential novel therapeutic approaches for treating CNS injury.

## Methods

### Animal and Surgical Procedures and Isolation of DNA for WGBS

Animal procedures were approved by the Institutional Animal Care and Use Committees (IACUC) of the University at Albany (optic nerve crush) and Morehead State University (spinal cord transection), in accordance with the National Institutes of Health Guide for the Care and Use of Laboratory Animals. All surgeries and dissections were performed on fully anesthetized animals (immersion in neutral-buffered 0.1% and 0.02–0.04% tricaine methanesulfonate (MS222, Sigma Aldrich) for juvenile frogs and tadpoles, respectively). For the sake of consistency, *Xenopus laevis* tadpoles and juvenile frogs were from an albino strain obtained from the same supplier (Xenopus Express, Brooksville, FL). Because DNA for WGBS was collected at the same time as the RNA used in an earlier study, we refer the reader to that paper for full details [11]. Briefly, the optic nerve of 1–3-month-old, post-metamorphic frogs (unsexed juveniles) was crushed at the orbit (ONC) and the spinal cords of juvenile frogs and NF stage (st.) 53 tadpoles [81] were transected (SCI) at the mid-thoracic level, as described [11, 36, 38, 130]. Eyes and hindbrains were dissected at a time coinciding with the peak of regenerative axon outgrowth – 7 days post SCI and 11 days post ONC. DNA for WGBS was isolated from the same animals as RNA for RNA-seq, using the RNA/DNA/Protein Purification Plus Kit (Norgen Biotek Corp, catalog #47700) [11]. Each biological replicate consisted of either five pooled hindbrains (SCI) or six eyes (ONC). Regenerative CNS samples for SCI were made from tadpole hindbrain and age-matched, unoperated controls, and regenerative CNS samples for ONC were made from the operated eyes of juvenile frogs, the contralateral unoperated eyes from the same animals, and control eyes from unoperated animals of the same age. For the sake of making fair comparisons between ONC and SCI, in the current study we relied on the eyes of unoperated frogs as the principal control group, unless specified otherwise, because for SCI, surgeries were necessarily performed on separate animals from controls. Non-regenerative CNS samples were made from frog SCI hindbrain and unoperated control hindbrains from frogs of the same age and cohort. Approximately 1 microgram of purified DNA from each of the 21 samples (3 replicates, 7 conditions) was subsequently shipped to the Genomics, Epigenomics and Sequencing Core at the University of Cincinnati for bisulfite WGBS.

### Whole genome bisulfite sequencing

To prepare the library for WGBS, first, 300 ng of intact genomic DNA quantified by Qubit assay (Thermo Fisher, Waltham, MA) was sheared by Covaris S2 focused-ultrasonicator (Covaris, Woburn, MA) to a peak size of 150-200 bp, and validated by 2100 Bioanalyzer High Sensitivity DNA assay (Agilent, Santa Clara, CA). Next, using the NEBNext Ultra DNA Library Prep Kit (New England BioLabs, Ipswich, MA), DNA fragments were end-repaired, 3’ end adenylated, and ligated to NEBNext Methylated Adaptors. The ligated DNA was then bisulfite-modified using an EZ DNA Methylation-Gold kit (Zymo, Irvine, CA), enriched and indexed by 8 cycles of PCR using Platinum Taq DNA Polymerase (Thermo Fisher) and NEBNext Index and Universal PCR primers. After AMPure XP bead purification (Beckman Coulter, Indianapolis, IN) and size selection of the indexed libraries, the quality of the libraries was assessed by Bioanalyzer. This analysis confirmed that all libraries were within the recommended size range, with no evidence of contamination; bisulfite conversion rates, monitored on human DNA processed with the same reagents at the facility were >98%. Finally, the library concentration was qPCR-quantified using an NEBNext Library Quant Kit (New England BioLabs) and QuantStudio 5 Real-Time PCR System (Thermo Fisher).

To generate sequencing data, the uniquely indexed, imbalanced WGBS libraries were pooled with well-balanced libraries to fill each lane of a flow cell for clustering in a cBot system (Illumina, San Diego, CA). The pooled libraries at the final concentration of 15 pM were clustered onto a flow cell using Illumina TruSeq PE Cluster kit v3 and sequenced under paired-end conditions at 2X101 bp, using a TruSeq SBS kit v3 on an Illumina HiSeq 1000 system, according to the Illumina recommended protocol to a nominal depth of 15× genome coverage (14.8 ± 0.3× (SE)). Four sequencing runs were performed, with Q30 scores of 94.35%, 91.33%, 88.39%, and 90.27% (91.09 ± 1.25%, mean ± SE), which were all above the expected 85% of bases higher than Q30 at 2×100 bp. The 42 paired-end FASTQ sequence files were further analyzed for quality by FastQC (Babraham Bioinformatics), which confirmed the high quality of the sequences (lowest median Phred score at any position in the sequence averaged 32.9 ± 0.3 (95% CI; N = 42).

### WGBS Read Alignment and Differential Methylation Analysis

Sequencing reads were aligned to the *Xenopus laevis* genome (version 9.1 [122]; downloaded from http://www.xenbase.org RRID:SCR_003280 [16, 47, 52]) using Bismark (version 0.18.2 [56]). Alignments were performed using the default parameters on untrimmed pairwise alignments, except for the more relaxed scoring parameters needed to align bisulfite sequences (*--score_min L,0,-0.6*) in Bowtie2 (v2.2.9 [61]). The resulting mapping efficiencies stayed within the requisite range of 75-80% unique hits (77.0 ± 0.9% (SE)), with duplicate alignments constituting another 6-8% of the total (7.2 ± 0.1% (SE)). As discussed elsewhere [11], reads initially flagged as potentially duplicate alignments can occur in *Xenopus laevis* due to its ancestrally (~ 30 Mya) duplicated genome and high levels of repetitive sequence (25 – 30%) [98]. Potential duplicate alignments could be resolved for the vast majority by using the alignment with the higher score to assign them to S or L homeologs. Deduplication in Bismark classified 4.5 ± 1.8% (95% CI) of sequences as duplicate reads. The Bismark methylation extractor script (with *--paired-end* and *–comprehensive* options) was run on each of the unfiltered alignments to produce the methylation coverage files in CpG, CHH, and CHG contexts. The overall methylation rate for all seven conditions, in all three contexts was 88.5 ± 5.1% (95% CI; N=7), which compared favorably with a previously reported rate of 92% for gastrula stage *X. laevis* embryos [29]. The external module “bismark2bedGraph” was then used to convert these files into the bedGraph format, which in turn were transformed into bigWig files using the script *BedGraphToBigWig v.4* with default parameters (http://hgdownload.cse.ucsc.edu/admin/exe/). The bigWig score files were used for direct observation in Integrative Genomics Viewer (IGV) v2.3.88 [96, 110] and also as an input for generating the heatmaps. The differential methylation tracks in IGV (log_2_(fold-change)) were generated with the program *bigwigCompare* from the deepTools2.0 package [92].

Analysis comparing tissue- and injury-related differences in CpG methylation across promoter regions was performed using DMRfinder (version 0.3 [32]. In the first stage of this analysis, the *extract_CpG_data.py* script was used to convert the output from Bismark's aligner into a table of methylated/unmethylated counts at each CpG site, merging data from both strands. Next, the converted data from triplicates of matched samples (*i.e.*, injured vs. control, or pairs of tissues) were combined using the *combine_CpG_sites.py* script, thereby clustering methylation counts at individual CpG sites into genomic regions. For the final stage of this analysis, the DMRfinder script *findDMRs.r* conducted pairwise tests of sample groups of triplicates to find genomic regions that were differentially methylated. The underlying statistics are based on the beta-binomial hierarchical modeling and Wald test implemented in the Bioconductor package DSS [30]. Default program parameters were used except for the *–t 0* option, which allowed us to collect information on all the DMRs reported, regardless of methylation differences, p and FDR values. This allowed us to perform a further filtering step. For this final filtering step, we first used the set of CpG DMRs from a given pairwise comparison to determine empirically the optimal FDR for removing false positives from data by using the distribution of DMRs for p > 0.3 ([12, 53, 54]; also see https://www.nonlinear.com/progenesis/qi-for-proteomics/v1.0/faq/pq-values.aspx). This empirically determined, optimal FDR was then used to further filter DMR data from the p < 0.05 subset, resulting in the final list of DMRs for a given pairwise comparison. The generated lists of DMRs were then combined with position-specific information on corresponding gene coordinates and their annotations, predicted promoter regions, and matching data on RNA expression differences, using the utility *bedtools intersect* from the BEDtools suite v2.27.0 [90]. To identify DMRs that overlapped with promoter regions, DMRs were selected that fell within the region between 750 bp upstream and 250 bp downstream of predicted transcription start sites (TSS), which were based on gene models from the primary transcript genomic annotation file (XL_9.1_v1.8.3.2.primaryTranscripts.gff3; Xenbase v9.1 at http://www.xenbase.org). RNA expression data for this analysis came from an earlier study performed on the same animals [11], analyzed by CuffDiff2 (v.2.2.1) [GSE 137844] [114, 115]. To view RNA expression differences in IGV, the RNA-seq alignment files for corresponding tissues were used to generate RNA expression and differential expression bigwig score, using the *bam2bigwig* script (https://github.com/lpryszcz/bin/blob/master/bam2bigwig.py) and the *bamCompare* utility of deepTools2.0.

The heatmaps demonstrating spatial correlations between DNA methylation and RNA expression levels were generated using the *computeMatrix* and *plotHeatmap* modules of deepTools2 and a bin size of 50 bp. These modules were run using the default parameters (except for the color choices) using the bigwig score tracks and .bed files for the regions that were plotted, which were, generated either by partitioning the RNAseq expression values [11] into four quartiles, or by using the gene coordinates for hyper- or hypo-methylated DMRs.

Gene Ontology (GO) term enrichment analysis was performed using Metascape (v3.0) [116]. Briefly, lists of genes that had injury-induced CpG DMRs near the TSS

and were significantly (FDR < 0.05) differentially expressed were analyzed separately for each methylation/expression state and injury condition. L and S homeologs were combined under a single human gene symbol and searched for membership in ontology groups across all species. Genes in Fig, 8 comprised 738 up-regulated and 460 down-regulated genes undergoing increased injury-induced methylation in tadpole hindbrain, derived from 1853 genes undergoing both differential, injury-induced methylation and RNA expression in any tissue. Only the top 20 GO terms are presented.

### ChIP-seq and 5hmC DIP-seq

Chromatin immunoprecipitation-sequencing (ChIP-seq) was performed in duplicate on newly prepared samples representing all seven conditions used for WGBS and pooled similarly, albeit necessarily from different animals. Samples for ChIP-seq were prepared using the Manual iDEAL ChIP-seq kit for histones (Diagenode #C1010051) according to the manufacturer’s protocols, and the following antibodies: H3K4me3 (Abcam ab8580; lot GR3201240-1), H3K27ac (Abcam ab4729; lot GR3205523-1). Dissected tissues were frozen immediately on dry ice and stored at -80°C for later processing. Tissues were thawed while homogenizing them in PBS containing protease inhibitor cocktail, in 2-ml Pyrex glass homogenizers. After low-speed centrifugation (1,300 rpm for 5 min at 4°C), pellets were resuspended and cross-linked in PBS with 1% formaldehyde for 10 min at room temperature. Cross-linking reactions were quenched with glycine. Samples were subsequently prepared for lysis and sonication in a Diagenode Standard Bioruptor (Chromatin Shearing and Optimization kit, Diagenode #C01020010). Sonication was performed at the high-power setting for 30 cycles of 30 seconds on and 30 seconds off. This was repeated until the sheared chromatin was 150-350 bp in size, as verified by gel electrophoresis. Immunoprecipitation was done using 5 μg of antibody and 50 μl of DiaMag protein A-coated magnetic beads. Barcoded DNA libraries were prepared from the immunoprecipitated DNA, as well as from equal amounts of pre-IP DNA from sheared chromatin, using the NEBNext Ultra II DNA Library Prep kit for Illumina sequencing. Sequencing (Illumina NextSeq500) was performed at the University at Albany Center for Functional Genomics core facility (75 bp, single end reads).

DNA immunoprecipitation-sequencing for 5hmC (5hmC DIP-seq) was performed on genomic DNA purified from ONC samples (operated eyes, contralateral unoperated eyes, control eyes from unoperated animals), prepared as described for WGBS. Genomic DNA (1 μg each) was sonicated for 14 cycles of 15 seconds on and 90 seconds off, using a Diagenode Standard Bioruptor to produce fragments of 200–600 bp in length, which was verified by gel electrophoresis. The sheared DNA was then subjected to 5hmC-IP using the kit’s 5hmC antibody and its non-specific IgG control antibody, overnight at 4°C, according to the manufacturer's instructions (hMeDIP Kit, Diagenode #C02010031, mouse monoclonal mAb). The immunoprecipitated DNA was washed and released from the antibody complex by proteinase-K digestion and resuspended in 100 μl of DNA-IP buffer (DIB) provided by the kit. Because of low IP yields, replicate DNAs were necessarily pooled for library preparation and sequencing, which was performed as for ChIP-seq

The quality of the FASTQ files containing the sequenced reads was first checked using FastQC [3] (https://www.bioinformatics.babraham.ac.uk/projects/fastqc/). Adapter sequences and barcodes were removed using Trimmomatic [15] and FastX Tookit, and low-quality reads (quality score Q < 30) were filtered from the data using Trim Galore! v0.3.7 (https://www.bioinformatics.babraham.ac.uk/projects/trim_galore) with Cutadapt v1.9 [76]. Read alignments were performed against the *Xenopus laevis* genome (v.9.1) using Bowtie2 (v2.2.9), with the *--very-sensitive* preset option. For ChIP-seq, the total numbers of successfully aligned reads averaged as follows (million base pairs ± SE): H3K4me3, 53.2 ± 4.0; H3K27ac, 52.7 ± 3.7; Histone ChIP Input controls, 135.7 ± 8.9. For 5hmC DIP-seq, final numbers of aligned sequences (millions of base pairs ± SE) averaged 29.8 ± 2.5 for 5hmC IPs and 0.4 ± 0.06 for control IPs (the numbers for control IgG IPs were low because the control antibody precipitated very little DNA, as expected). The generated BAM alignment files were converted into the bigWig score files using *bam2bigwig*. Heatmaps were generated as described for bisulfite WGBS data.

### Data Availability

FASTQ and bigWig files are available at the Gene Expression Omnibus (GEO) repository [https://www.ncbi.nlm.nih.gov/gds] under accession number GSE183357. RNA-seq data from our previous study [11] is available at GEO (GSE137844).

## Supplementary Information

**Additional file 1.** Excel file containing tabulated correlations between TSS-associated, CpG DMRs and RNA expression. Detailed legends are provided within the file, Tab 1. Data are in Tab 2 (Supplementary Table 1). Tab 3 contains Supplementary Table 2, which is the Chi-squared 2×2 correlation table.

## Data Availability

The datasets generated and/or analyzed during the current study are either available in the Gene Expression Omnibus (GEO) repository (GSE183357) *[**https://www.ncbi.nlm.nih.gov/gds**]* or included in this published article and its supplementary information files. RNA-seq expression data from Belrose et al. [11] are available from GEO (accession number GSE137844). Genomic resources for *Xenopus laevis* are available from Xenbase (http://www.xenbase.org, RRID:SCR_003280

## Abbreviations

5mC: 5-methylcytosine
5hmC: 5-hydroxymethylcytosine
Chr: Chromosome
ChIP-seq: Chromatin-immunoprecipitation sequencing
CNS: Central nervous system
DESR: Differentially expressed in successful regeneration
DIP-seq: DNA-immunoprecipitation sequencing
FDR: False discovery rate
FPKM: Fragment per kilobase million
IGV: Integrative Genomics Viewer
LSD: Least significant difference
MS: Methylation sequencing
ONC: Optic nerve crush
PNS: Peripheral nervous system;
RNA-seq: RNA sequencing
SCI: Spinal cord injury
WGMS: Whole Genome Bisulfite Sequencing
*X. laevis*: *Xenopus laevis*
